# Oral administration of *Lactobacillus plantarum* 299v modulates gene expression in the ileum of pigs: prediction of crosstalk between intestinal immune cells and sub-mucosal adipocytes

**DOI:** 10.1007/s12263-015-0461-7

**Published:** 2015-04-11

**Authors:** Marcel Hulst, Gabriele Gross, Yaping Liu, Arjan Hoekman, Theo Niewold, Jan van der Meulen, Mari Smits

**Affiliations:** Animal Breeding and Genomics Centre, Animal Sciences Group of Wageningen UR, P.O. Box 338, 6700 AH Wageningen, The Netherlands; Nutrition and Health, Katholieke Universiteit Leuven, Kasteelpark Arenberg 30, 3001 Heverlee, Belgium; Central Veterinary Institute, Animal Sciences Group of Wageningen UR, P.O. Box 65, 8200 AB Lelystad, The Netherlands; Mead Johnson Nutrition, Nijmegen, The Netherlands; USC Epigenome Center, University of Southern California, Los Angeles, CA USA

**Keywords:** *Lactobacillus plantarum* 299v, Pigs, Intestine, Gene expression, Crosstalk

## Abstract

To study host–probiotic interactions in parts of the intestine only accessible in humans by surgery (jejunum, ileum and colon), pigs were used as model for humans. Groups of eight 6-week-old pigs were repeatedly orally administered with 5 × 10^12^ CFU *Lactobacillus plantarum* 299v (*L. plantarum* 299v) or PBS, starting with a single dose followed by three consecutive daily dosings 10 days later. Gene expression was assessed with pooled RNA samples isolated from jejunum, ileum and colon scrapings of the eight pigs per group using Affymetrix porcine microarrays. Comparison of gene expression profiles recorded from *L. plantarum* 299v-treated pigs with PBS-treated pigs indicated that *L. plantarum* 299v affected metabolic and immunological processes, particularly in the ileum. A higher expression level of several B cell-specific transcription factors/regulators was observed, suggesting that an influx of B cells from the periphery to the ileum and/or the proliferation of progenitor B cells to IgA-committed plasma cells in the Peyer’s patches of the ileum was stimulated. Genes coding for enzymes that metabolize leukotriene B4, 1,25-dihydroxyvitamin D3 and steroids were regulated in the ileum. Bioinformatics analysis predicted that these metabolites may play a role in the crosstalk between intestinal immune cells and sub-mucosal adipocytes. Together with regulation of genes that repress NFKB- and PPARG-mediated transcription, this crosstalk may contribute to tempering of inflammatory reactions. Furthermore, the enzyme adenosine deaminase, responsible for the breakdown of the anti-inflammatory mediator adenosine, was strongly down-regulated in response to *L. plantarum* 299v. This suggested that *L. plantarum* 299v-regulated production of adenosine by immune cells like regulatory T cells may also be a mechanism that tempers inflammation in the ileum, and perhaps also in other parts of the pig’s body.

**Electronic supplementary material:**

The online version of this article (doi:10.1007/s12263-015-0461-7) contains supplementary material, which is available to authorized users.

## Introduction

Probiotic bacteria may exert a beneficial effect on the host’s health by steering immunological reactions in the gastrointestinal (GI) tract. One example for a bacterial strain that is applied in fermented probiotic drinks is *Lactobacillus plantarum* (*L. plantarum*: De Vries et al. 2006; Molin 2001). *L. plantarum* originates from healthy human colonic mucosa and belongs to the predominant mucosal *Lactobacillus* flora in the human intestine and survives in gastrointestinal passage (Johansson et al. 1993; Molin 2001). However, knowledge about the mechanisms underlying protective effects of probiotic bacteria like *Lactobacilli* in the complex environment of the GI tract is still limited (reviewed in reference, van der Meulen et al. 2010).

In gut mucosa of mammals, specialized immunological cells (dendritic cells, M cells, macrophages, etc.) constantly survey the lumen for the presence of potential pathogenic microorganisms. Conserved non-self molecular structures/motifs common for groups of microbes (microbe-associated molecular patterns, MAMP) are recognized by toll-like receptors (TLRs) and pattern recognition receptors (PRRs) of these immune cells. Recognition by TLR–PRRs discriminates between MAMP of pathogenic microbes (pathogen-associated molecular patterns, PAMP) and of commensal bacteria that assist in the fermentation and absorption of food. While PAMP activates TLR-mediated innate defence mechanism and a pro-inflammatory epithelial response to protect the host from infection, recognition of MAMP of commensal bacteria by TLR’s is ignored and prevents a pro-inflammatory response. For probiotic bacterial species, several surface structures and molecules (e.g. flagellins, polysaccharides and lipoteichoic acids) are identified as MAMP (Lebeer et al. 2010). Similarly, as was observed for commensal and pathogenic bacteria, small structural variations between MAMP of different probiotic bacterial species determine the type of response mediated by TLR–PRR activation (reviewed Lebeer et al. 2010). To understand the complex mechanisms how these MAMP of probiotic bacteria interact with cells of the gut, in vivo studies are needed in which heterogeneous multi-cellular systems interact mutually with microorganisms. Such in vivo studies will provide knowledge about how probiotic bacteria exert their beneficial effects in the gut, and how induced (immune) responses in the mucosa are transmitted to other parts of the host body to improve health.

In humans, transcriptional changes in duodenal mucosa upon continuous intraduodenal infusion of *L. plantarum* strain WCFS1 for 6 h have been studied by collecting duodenal biopsies (van Baarlen et al. 2009). Due to its safety and survival in the human gastrointestinal tract and the availability of the complete genome sequence, strain WCFS1 is particularly suitable to explore probiotic modes of action on the molecular level in humans (De Vries et al. 2006; van Baarlen et al. 2009). However, such studies are invasive for volunteers and only allow sampling in the duodenum and distal colon and not in parts of the intestine like the ileum and jejunum. With regard to digestion of food and other physiological functions, among all mammals, the intestine of pigs probably best resembles that of humans (van der Meulen et al. 2010). Therefore, in the current study, we investigated host–microbe interactions in the jejunum, ileum and colon of pigs after repeated oral administrations of *L. plantarum* 299v, starting with a single dose followed by three consecutive daily dosings 10 days later. For assessment of the transcriptional response in the mucosa of these intestinal tissues, gene expression profiles were generated using porcine whole-genome microarrays and compared with those obtained from pigs administered with PBS. The results of this study showed that *L. plantarum* 299v modulates in vivo transcriptional profiles mainly in the ileum. Bioinformatics analysis of response genes enabled us to generate a predictive overview of processes/pathways activated on a transcriptional level in the ileum after repeated consumption of *L. plantarum* 299v.

## Methods

### Animal experiment

The animal experiment was approved by the institutional animal experiment committee “Dier Experimenten Commissie Lelystad” (DEC Protocol 2006110.c) in accordance with the Dutch regulations on animal experiments. Weaned male 4-week-old pigs (Duroc x Topigs 20; eight per group) were obtained from a commercial Dutch piggery. Starting directly after weaning until the end of the experiment, animals were fed ad libitum with commercial dry pig weaning feed. After 14 days of adaptation to housing conditions, faecal samples were collected and the experiment started on the next day (day 0) with a single oral administration of bacterial suspensions to the animals: *L. plantarum* 299v wild-type strain (Johansson et al. 1993), approximately 1 × 10^12^ colony-forming units (CFU) per ml in phosphate-buffered saline (PBS), or PBS as a negative control (each 5 ml). Using a syringe, the suspension was injected in the oral cavity of the piglets. After a period of 10 days during which faecal samples of the animals were collected for microbiological analysis, the treatment was repeated for three consecutive days. Twelve hours after the last oral administration, the animals were euthanized by barbiturate overdose. Mucosal scrapings were collected at the mid of the jejunum, ileum and colon (not rinsed), immediately frozen in liquid nitrogen, and stored at −80 °C until total RNA was extracted.

### Bacterial strains and culture media

For the animal experiment, a rifampicin-resistant derivative of the wild-type *L. plantarum* 299v strain was selected as described earlier (Johansson et al. 1993; Bron et al. 2004; Gross et al. 2008). *L. plantarum* 299v was grown overnight at 37 °C in Man Rogosa Sharp (MRS) broth (Merck, Darmstadt, Germany) containing 100 μg/ml rifampicin. Bacterial suspensions in PBS were prepared by 2 rounds of centrifugation of the bacterial culture and washing/re-suspending of the bacterial pellet in PBS. Bacterial counts of the *L. plantarum* 299v suspensions in PBS administered to the pigs were determined by plating of serial dilutions on appropriate agar plates. Faecal samples of 1 cm^2^ were homogenized in physiological salt solution and stored at 4 °C before serial dilutions of these samples were plated on appropriate agar plates. Samples of 1 cm^2^ frozen mucosal scrapings collected from the jejunum, ileum and colon were homogenized in PBS using the TisuPrep Homogenizer Omni TP TH220P and stored at 4 °C before serial dilutions of these samples were prepared and plated on appropriate agar plates. Serial dilutions of homogenized faecal and mucosal scrapings were plated on MRS agar containing 100 μg/ml rifampicin and incubated anaerobically at 37 °C for 48 h before CFU were counted. Results from bacterial counts were expressed as mean log10 CFU/g faecal sample ± standard error of the mean (SEM).

### Isolation of total RNA and microarray analysis

Total RNA was isolated from approximately 0.5 g of frozen mucosal scrapings collected from jejunum, ileum and colon of individual pigs. The tissue was homogenized in 2 ml TRIzol reagent (Invitrogen, Breda, the Netherlands), and the extractions were essentially performed according to the instructions of the manufacturer with additional steps to remove proteoglycan and polysaccharide contaminations and DNAse treatment as described earlier (Niewold et al. 2005). Finally, RNA was dissolved in RNAse-free water and stored at −80 °C until further use. All samples were checked for RNA integrity by agarose gel electrophoresis and UV-spectrophotometry.

After additional RNA clean-up (NucleoSpin RNA II, Macherey–Nagel, Düren, Germany), the quality and integrity of the samples were analysed using Agilent Lab-on-a-Chip and Bioanalyzer (Agilent Technologies, Amstelveen, the Netherlands). All samples scored a RNA integrity number (RIN value) of ≥9. Samples from the same intestinal location of individual animals of the same treatment group were pooled by mixing equal amounts of RNA (*n* = 8 for all groups, except for the jejunum PBS control: *n* = 7). Quality of the RNA pools was assessed again, all scoring a RIN value of ≥9. For gene expression analysis, Affymetrix Porcine Genome Microarray Chips (Affymetrix, Santa Clara, CA) were used, following Affymetrix protocols for hybridization, washing, staining and scanning of the arrays. Briefly, biotin-labelled cRNA was synthesized from mRNA by Oligo(dT)-priming with the Affymetrix One-Cycle Target Labeling and Control Kit using 2 μg of pooled total RNA as input. 12.5–20 μg cRNA was further fragmented, and finally, 10 μg was hybridized to the arrays. Quality control and pooling of the RNA samples, Affymetrix microarray analysis and quality control of the arrays were performed externally by ServiceXS (Leiden, the Netherlands). Also data processing, array normalization and analysis of the array data were performed at Service XS using GeneSpring Software (Agilent Technologies). Briefly, spots with a raw intensity ≤50 were filtered out, and a 50th percentile normalization was performed for each chip. In addition, for each individual probe/gene, the intensity was normalized using the median intensity calculated from all chips/samples. The expression profiles induced by the treatment with *L. plantarum* 299v were compared with the PBS (control) treatment separately for the jejunum, ileum and colon. A threshold level of >2× and <0.5× fold change (FC) of differential expressed probes was used. Normalized array data were posted in the EMBL-EBI ArrayExpress database under deposit number E-MEXP-2198. Note that the results presented in this manuscript were part of a larger study. In E-MEXP-2198 sources, MSA 09, 13, and 17 represent the array data of PBS RNA pools of the jejunum, ileum and colon, respectively, and sources MSA 10, 14, and 18 represent the *L. plantarum* 299v wild-type RNA pools of the jejunum, ileum and colon, respectively.

### Quantitative PCR

The relative concentration of interleukin-1-beta (IL1B) and carbonic anhydrase 2 (CA2) mRNA in all individual RNA samples extracted from mucosal scrapings from the ileum was determined by quantitative real-time PCR (QRT-PCR). RT reactions were performed with Superscript III (Invitrogen) and random hexamer primers (pdN6) according to the manufacturer’s instructions using 250 ng of RNA template. The gene-specific primers and cycle specifications for quantification of IL1B cDNA were applied as described recently (Schreur et al. 2011). Gene-specific primers for CA2 were developed from the pig mRNA sequence XM_001927805.1. A CA2-specific cDNA fragment of 127 bp was amplified using a 20-mer forward (CAACGGCCACTCTTTCAACG) and reverse primer (TTGCCCATCAGATGAACCCC), using the same cycle protocol as described for IL1B (Schreur et al. 2011). The quantity of 18S rRNA in each RNA sample was determined by QRT-PCR using the above described RT reaction products (Durand et al. 2009) and used to normalize IL1B and CA2 data. The quantity of 18S ribosomal RNA showed no essential differences among all individual RNA samples extracted from scrapings.

### Bioinformatics and functional analysis

In addition to the annotation provided by Affymetrix, oligonucleotide sequences of differentially expressed probes not annotated yet, or annotated as Unigene, tentative consensus sequences (TC) or mRNA accession number, were compared with the NCBI non-redundant nucleotide databases using blastn to assign a gene name to these probes. Probes that did not produce a significant match with any other eukaryotic mRNA/gene were excluded from gene lists used for functional analysis. Throughout this manuscript, official human gene symbols (HUGO Gene Nomenclature Committee: http://www.genenames.org) were used in the text and in all (supplementary) figures and tables. In all results, paragraphs beneath, information about the biological functions of genes was retrieved by consulting the “GeneCards” (Weizmann Institute of Science) and the NCBI Gene reports, and literature linked to these reports. For genes with multiple functions, mostly only information is provided about their function related to processes/pathways identified in this study, and we refer for information about additional functions to these reports.

The Database for Annotation, Visualization and Integrated Discovery (DAVID version 6.7) website (da Huang et al. 2009) and the “Set Distiller” module of GeneDecks (Stelzer et al. 2009) were used to assign genes to a specific pathway. Because far more human genes are annotated and more information in databases is available for humans than for pigs, the human background was used for this functional analysis. From DAVID, pathways (KEGG) with a *p* value of <0.1 (EASE score) were retrieved. In GeneDecks, pathways (KEGG, MLPR, CST, GeneGlobe Pathway Central, Invitrogen and Ingenuity) were called significant with a *p* value <0.05 using the Set Distiller algorithm. KEGG pathways retrieved from DAVID were only listed when not called significant by GeneDecks, or in case more genes were listed by DAVID than by GeneDecks. Pathways that contained <5 regulated genes were not retrieved. Associations of genes with chemical compounds with immune-modulating properties were retrieved from GeneDecks (*p* value of <0.05). Using the “tissue expression” module of DAVID, regulated genes were explored for expression in specific (subsets of) immune cells and organs by enrichment analysis using the GNF_U133A_QUARTILE cDNA libraries (http://biogps.org/#goto=welcome). Cell-specific libraries enriched with a *p* value of <0.05 were considered significant. From DAVID, “Functional Annotation charts”, transcription factors or genes involved in regulation of transcription were identified by gene-ontology analysis and uploaded as sub-list in GNCPro (free online software developed and maintained by SABiosciences Inc. a Qiagen company) to establish associations between these genes (i.e. to build a network of transcription factors and regulators). Using the protein interaction tool of DAVID, regulated gene sets were enriched for specific transcription factor binding sequences (UCSC_TFBS module). Seven transcription factors for which the enrichment analysis of their corresponding transcription recognition sequence was most significant (*p* value of <0.05) were added to the DAVID sub-list loaded in GNCPro. Non-interacting genes were omitted from the displayed network.

Functional associations between immune-modulating chemicals, proteins encoded by differentially expressed genes and enzyme substrates/products linked to these proteins were established using the (protein)–protein–chemical interaction web tool STITCH 4.0 beta (Kuhn et al. 2009). Relevant chemicals were uploaded together with list of genes in STITCH. Associations with a confidence score of ≥0.4 (medium level) were selected from output files and displayed in a network. In supplementary file Table S3, the type and confidence level of each association are listed.

### Statistical analysis

When appropriate, results are indicated ± SE of the mean (SEM) and the significance of the difference between results of the treatment and control conditions was calculated using Student’s *t* test (two sided; considered statistically significant when *p* is <0.05).

## Results

### *L. plantarum* persistence characteristics in the pig intestine

**Fig. 1:**
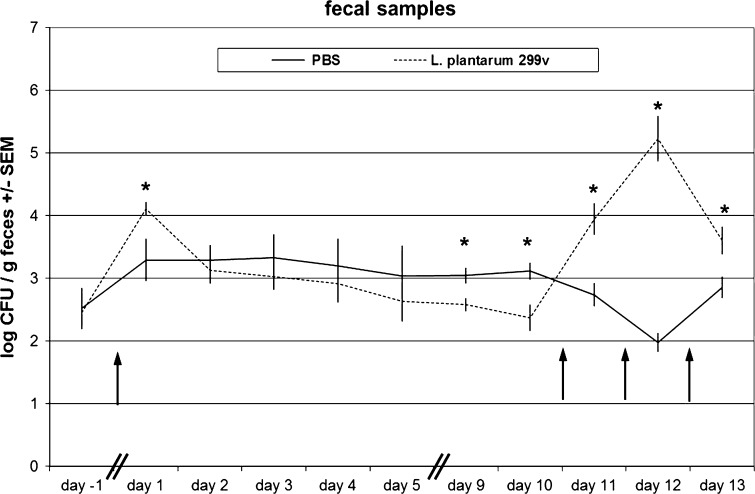
Average faecal excretion of rifampicin-resistant bacterial counts (expressed in log10 CFU ± SEM *L. plantarum*/g faecal sample) per experimental group of animals (*n* = 8), either administered with PBS (control; *black line*), or *L. plantarum* 299v (*dotted line*). *Arrows* indicate the time points of oral administration of bacterial suspensions (at the beginning of day 0, and at the end of day 10, 11 and 12). *Significantly different from PBS group (*t* test *p* < 0.05)

Frequent delivery of a *L. plantarum* 299v suspension in the oral cavity (e.g. 3 times a day over a period of several weeks) would be too stressful for young piglets. Therefore, we decided to administer a 10- to 100-fold higher dose compared with a dose (10^10^ to 10^11^ CFU, e.g. see Mangell et al. 2012) normally given to human cohorts in a clinical trial and consumed before each meal and over a period of several weeks. After the first oral administration, higher levels of rifampicin-resistant bacteria were found on day 1 in faecal samples of treated pigs compared with PBS-treated pigs (Fig. 1). After that, bacterial counts rapidly descended to a similar level as was observed for PBS-treated pigs, suggesting that no significant colonization of *L. plantarum* 299v took place in the GI tract of pigs and that the survival rate of *L. plantarum* 299v in the GI tract is low. To ensure that all parts of the intestinal mucosa became sufficiently exposed to *L. plantarum,* oral administration was repeated on 3 consecutive days (day 10, 11 and 12; Fig. 1). Twelve hours after the last administration (day 13), and just before the pigs were euthanized and mucosal samples were collected, again the level of rifampicin-resistant bacteria in faecal samples was significantly higher (*p* < 0.05) in *L. plantarum* 299v-treated pigs compared with PBS-treated control animals. However, bacterial counts from plating of the serial dilutions of homogenized mucosal scrapings were below the detection limit (approximately 2 log_10_ CFU/cm^2^ of scrapping) for all jejunal, ileal and colon scraping in all experimental groups (data not shown). Again, this suggested that no significant colonization of *L. plantarum* 299v had occurred in the intestines of pigs.

### Gene expression patterns induced by *L. plantarum*

**Fig. 2:**
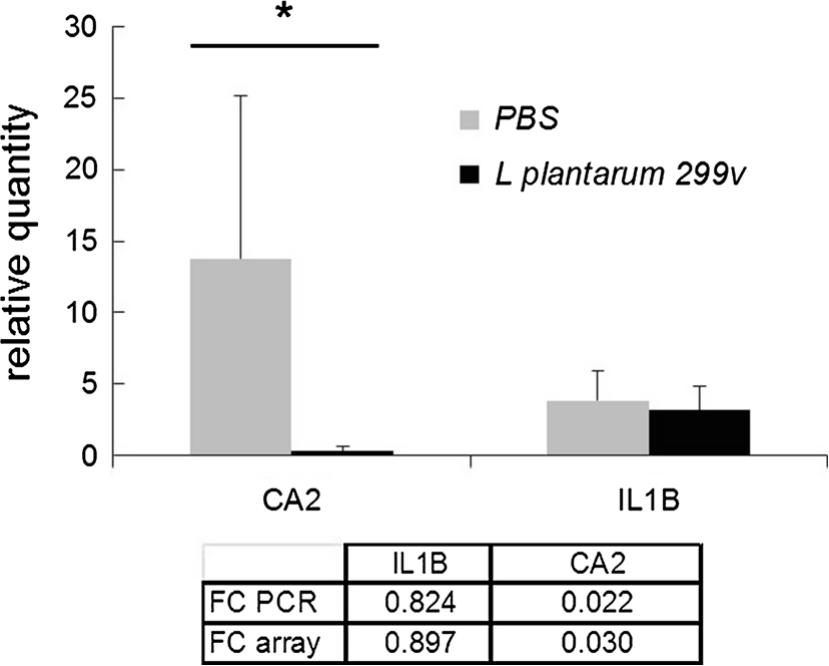
Quantification of IL1B and CA2 mRNA by QRT-PCR. Graph: Average of the relative concentration of mRNA measured in the ileum of pigs (*n* = 8) treated with *L. plantarum* 299v or treated with PBS. *Error bars* represent the STDEV (*n* = 8). Table: ratio of expression (FC, *L. plantarum* over PBS) measured on the microarray and by PCR. *Significantly different from PBS group (*t* test *p* < 0.05)

Gene expression patterns in jejunum, ileum and colon of pigs were investigated after repeated oral administration of *L. plantarum* 299v and compared with patterns from control pigs treated with PBS. Microarray analysis of the ileum detected a total of 463 probes hybridizing significantly different (FC >2 and <0.5) for *L. plantarum* 299v-treated pigs than for PBS pigs (listed in supplementary Table S1). These probes represented 407 unique mRNAs, from which 104 were down-regulated and 303 were up-regulated. For 52 differentially expressed mRNAs, two or three replicate probes, encoding a different part of the mRNA, were present on the array. No large differences in FC were measured between replicate/triplicates probes for 51 out of 52 differentially expressed mRNAs. In addition, FC values measured by microarray analysis were compared with FC values calculated from relative concentrations of IL1B and CA2 mRNA measured by QRT-PCR in individual RNA samples isolated from the ileum of all pigs (Fig. 2). The results of this QRT-PCR analysis confirmed the lower concentration of CA2 mRNA and nearly equal concentration of IL1B mRNA in *L. plantarum* 299v-treated compared with control animals, as was indicated by microarray analysis. Together with the consistent hybridization of replicate probes, QRT-PCR analysis on individual samples confirmed the microarray data that were assessed with RNA pools. However, the relatively large variation in CA2 mRNA concentration measured in the 8 RNA samples of pigs within the PBS group (Fig. 2) suggests that a considerable biological/genetic variation in expression levels of genes exists between individual pigs (see “Discussion”).

Compared with the ileum, a significantly lower number of differentially expressed genes affected by *L. plantarum* 299v were detected in the jejunum and colon, i.e. a total of 54 and 125 (up and down), respectively. To gain insight in the processes induced by *L. plantarum* 299v, sets of differentially expressed genes were analysed in DAVID and GeneDecks to retrieve significant pathways for each part of the intestine. In contrast to the ileum (see below), only one significant immune-related pathway (PPAR signalling) could be retrieved from DAVID (EASE score <0.1) and GeneDecks (*p* value of <0.05) for the colon, and none for the jejunum. Only 12 out of the 407 genes regulated in the ileum were also found regulated in the colon. Two of these genes were part of the PPAR signalling pathway (APOC3 and FABP2), and only 2 genes showed a defined function associated with immune cells (CCL25 and CR2). Regulation of several apolipoproteins in the colon (APOA4, APOB, APOA1 and APOC3) suggested that absorption of lipid vitamins and/or metabolism of lipids and lipoproteins was regulated in the colon. Clearly, these parts of the intestine showed a lower responsiveness towards *L. plantarum* 299v than the ileum. Therefore, further bioinformatics and functional analysis were conducted only for differentially expressed genes in the ileum.

### Pathway analysis of genes induced by *L. plantarum* 299v

Significantly regulated immunological and immune-related chemical/metabolic pathways affected by *L. plantarum* 299v administration were selected from DAVID and GeneDecks output files and are presented in supplementary Table S2 (down-regulated genes are shown bold and underlined). In Table 1, a selection of pathways is listed, providing a comprehensive overview of important processes in the ileum possibly induced by *L. plantarum* 299v. In addition, chemicals with immune-modulating properties showing a significant association with regulated genes (*p* value <0.05) were also retrieved from GeneDecks output files and displayed in Table S2.

**Table 1:** Pathway analysis (summarized)

Pathway	# genes	%	*p* value	Genes^a^
Cell cycle/checkpoint control	16	4.3	1.1E−16	ATR, BUB1B, CDC45, CDK2, KIF11, MCM2, MCM3, MSH2, MSH6, ORC1, PCNA, PRIM2, RCC2, TMPO, VRK1, WEE1
Cytoskeletal signalling	13	3.2	7.7E−11	**ACTG2**, **DES**, **GNB2L1**, LMNB1, MST4, **MYH10**, PFN1, PPP1R12A, RCC2, ROCK1, STMN1, TMPO, VAV1
Lymphocyte signalling	9	2.4	2.8E−09	BCL6, HCK, INPP5D, LCP1, **MECOM**, **NQO1**, PLCG2, PSIP1, SPI1
Cell cycle_Role of APC in cell cycle regulation	7	1.9	3.3E−09	BUB1, BUB1B, CCNB2, CDC27, CDK2, FBXO5, ORC1
Cell cycle_Transition and termination of DNA replication	6	1.6	1.4E−08	CDK2, LIG1, MCM2, PCNA, PRIM2, RFC3
Immune response_BCR pathway	7	1.9	3.1E−08	BCL6, CD79B, CR2, INPP5D, PIK3R1, PLCG2, VAV1
Chemokine signalling pathway (GeneDecks-KEGG)	8	2.1	2.7E−07	**CCL28**, CXCL13, CXCR4, HCK, PIK3R1, ROCK1, TIAM1, VAV1
Leucocyte transendothelial migration (GeneDecks-KEGG)	6	1.6	1.6E−06	**CDH5**, CXCR4, PIK3R1, PLCG2, ROCK1, VAV1
Focal adhesion (GeneDecks-KEGG)	7	1.9	3.2E−06	**CAPN2**, **COL1A1**, **COL3A1**, PIK3R1, PPP1R12A, ROCK1, VAV1
NFKB signalling Targets	5	1.3	8.2E−06	**AGT**, CD40, **CFB**, LTB, **NQO1**
hsa03030:DNA replication	6	1.7	1.7E−03	RFC3, LIG1, PRIM2, PCNA, MCM2, MCM3
hsa03430:Mismatch repair	5	1.4	2.1E−03	MSH6, RFC3, MSH2, LIG1, PCNA
hsa04662:B cell receptor signalling pathway	7	1.9	9.9E−03	CR2, PLCG2, PIK3AP1, CD79B, INPP5D, VAV1, PIK3R1
hsa00230:Purine metabolism	8	2.2	8.2E−02	**ENPP3**, RRM2, **AK1**, PRIM2, GUCY1B3, RRM2B, **PAPSS2**, **ADA**
hsa04666:Fc gamma R-mediated phagocytosis	8	1.9	2.9E−02	**ACTG2**, HCK, PLCG2, PIKFYVE, ARPC4, INPP5D, VAV1, PIK3R1
hsa04672:Intestinal immune network for IgA production	5	1.4	3.1E−02	CXCR4, **TNFRSF17**, **PIGR**, CD40, **CCL28**
hsa04115:p53 signalling pathway	5	1.4	8.5E−02	CCNB2, RRM2, RRM2B, ATR, CDK2
hsa03320:PPAR signalling pathway	5	1.4	8.9E−02	**HMGCS2**, **APOC3**, **PPARG**, **FABP2**, **MMP1**

DAVID pathways (hsa) with a *p* value of <0.1 (EASE score) and GeneDecks pathways with a *p* value of <0.05 were retrieved. The complete list of pathways is listed in supplementary Table S2. Official gene symbols are presented. Down-regulated genes are underlined and shown in bold

Genes mapped to the PPAR signalling pathway were all down-regulated by *L. plantarum* 299v; among them, the nuclear receptor PPARG which forms a receptor complex with the retinoic X receptor (RXR). Binding of ligands like 9-*cis*-retinoic acid (RA), fatty acids or eicosanoids to this complex activates adipocyte differentiation, lipid metabolism and negatively regulates macrophage activation. In conjunction with this, genes associated with steroids, leukotriene B4 and RA were called significant by GeneDecks (Table S2).

Several NFKB targets were up- or down-regulated in animals treated with *L. plantarum* 299v. CFB, AGT and NQO1 were down-regulated, and CD40 and LTB were up-regulated. AGT is a potent activator of cortisol by stimulating transcription factor nuclear receptor subfamily 4, group A (Romero et al. 2010). The Ba fragment of complement factor CFB (down-regulated here) inhibits the proliferation of pre-activated B-lymphocytes. NQO1 null-mice suffer from myeloid hyperplasia, suggesting that down-regulation of this quinone reductase supports lymphocyte proliferation. Binding of the LTB–LTA complex to the LT-beta receptor and binding of CD40 to its receptor on the surface of lymphocytes mediate TRAF-dependent NFKB signalling, which may initiate stimulation of lymphocyte attachment, B cell development/survival and T cell co-stimulation. The CD40 receptor also plays a role in the production of IgA in the intestine, a pathway called significant in this study. CCL28, a chemokine that may stimulate migration of IgA-committed plasma cells (B cells) from the Peyer’s patches to the lamina propia, and the Fc receptor PIGR, responsible for translocation of IgA complexes over the epithelial layer, were both down-regulated in response to *L. plantarum* 299v. In addition to the IgA pathway, B cell receptor signalling pathways were called significant, suggesting that an influx and/or proliferation of subsets of B cells was indeed regulated. Furthermore, pathway analysis suggested that phagocytosis (engulfment of antigens by macrophages, neutrophils, etc.) and activation/proliferation of NK cells were stimulated. The two most dominant pathways, cytoskeleton rearrangement and cell division were activated, most likely, to support the transformation of progenitor cells to active immune cells or to support their migration and homing.

The tissue expression module of DAVID predicted that a high percentage of regulated genes in the ileum are expressed in a lymph node library, CD4+ cells and CD14+ monocytes, suggesting that cell division in both lymphoid- and myeloid-derived progenitor cells was stimulated (see Table S2). The regulation of several B cell-specific markers/genes (e.g. CR2, CD40, EBF1, CXCR4, TNFRSF17, CXCL13, BCL6, BCL7A, CD79B, PIK3AP1, PIK3R1, UBE2V2), along with the calling of the IgA production pathway, suggests that this also applies for B cells. In addition, a high percentage of genes regulated by *L. plantarum* 299v were suggested to be expressed in adipocytes (Table S2). Except for FABP2, all down-regulated genes in the PPAR pathway were predicted to be expressed in adipocytes. PPARG itself and 9 out of the 20 genes associated with steroid metabolism were predicted to be expressed in CD14+ monocytes (shown in red in Table S2), whereas only 4 of them were predicted to be expressed in CD4+ T cells (shown in blue in Table S2).

### Transcriptional regulation of *L. plantarum* 299v-induced gene expression

**Fig. 3:**
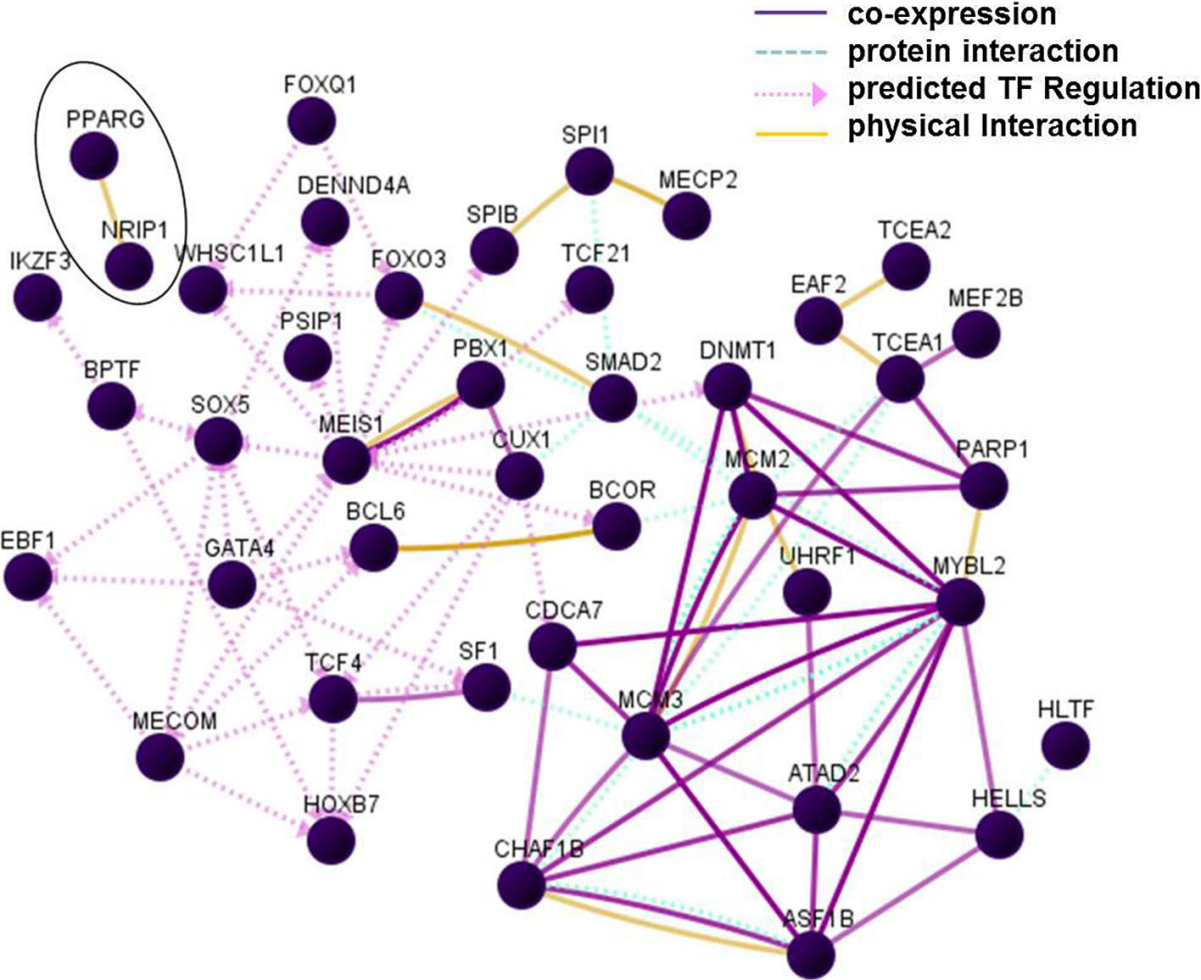
Interaction between transcription factors/regulators. Transcription factors added to the sub-list of transcription factors/regulators were: CUX1 [CDP], TRAF4 [CART1], PBX1, FOXQ1 [HFH1], SOX5, MEIS1 [MEIS1BHOXA9] and FOXO3A. The *abbreviations* used for these transcription factors/binding sites in literature and biological databases are listed between *squared brackets* after the official gene symbol. *TF* transcription factor

A sub-list of all up- and down-regulated transcription factors/regulators affected by *L. plantarum* 299v administration was retrieved from DAVID. In addition, using the UCSC_TFBS module of DAVID, an inventory of transcription factor-specific binding sites present in loci of regulated genes was performed (presented in supplementary Table S2). Transcription factors IKZF3 (alias LYF or Aiolos), GATA4, PPARG and MECOM, regulated in this study, were called significant in this analysis. Seven transcription factors, not regulated in this study, but for which binding sites were present in loci of a relatively high proportion of the here up- and down-regulated genes (see Table S2), were added to this sub-list. According to literature linked to GeneCards and NCBI Gene reports, transcription regulated by these “added” factors mediates differentiation/proliferation of immune cells. The supplemented sub-list was uploaded in GNCPro in order to build a network (Fig. 3). In the legend of Fig. 3, the added factors are listed. Most up-regulated transcription factors/regulators involved in the process of cell division (MCM2, MCM3, MYBL2, CHAF1B, ASF1B, ATAD2, SMAD2, TCEA1, DNMT1 and CDCA7) showed 4 or more associations with another factor. Up-regulated factors involved in immune-related processes were BCL6, BCOR, SPIB, SPI1 (B cell development), TCF4 (enhancer-regulator of immunoglobulin synthesis), and IKZ3 (alias Aiolos; regulates B cell activation and maturation to effector state), from which TCF4 showed more than 4 associations. Down-regulated factors with more than 4 associations were MECOM (involved in haematopoiesis) and GATA4. For PPARG, only one association was found, i.e. with NRIP1 (encircled in Fig. 3), a nuclear receptor co-repressor that binds to PPARG and can modulate transcriptional activation by the glucocorticoid receptor NR3C1. This suggests that down-regulation of PPARG and up-regulation of NRIP1 occurred in a different types of (immune) cell(s) than in the (progenitor) B cells, in which the majority of factors/regulators were predicted to act.

### Immune-modulatory compounds predicted to be associated with genes regulated by *L. plantarum* 299v

**Fig. 4:**
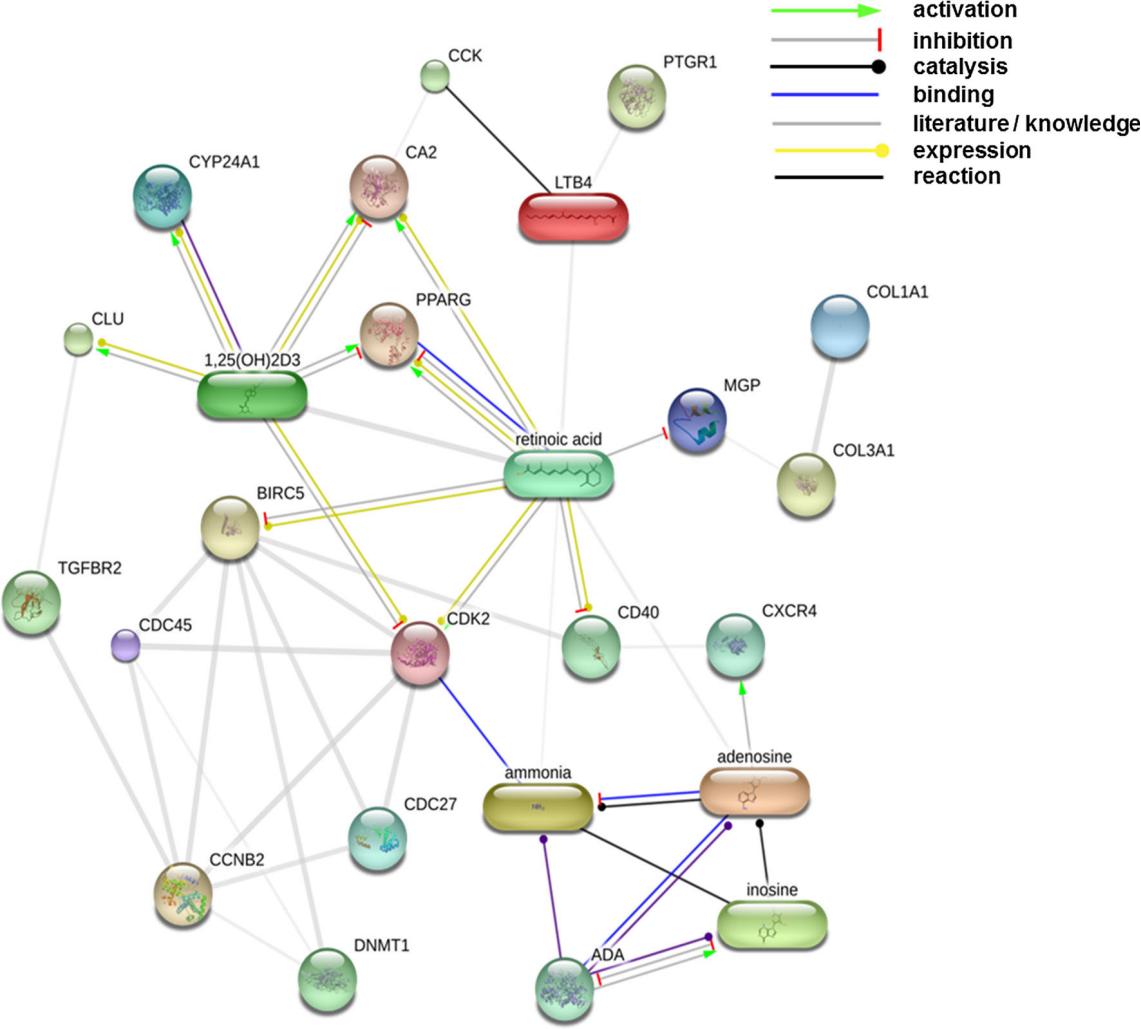
Interactions of leukotriene B4 (LTB4) , RA and Vitamin D3 with regulated genes. The displayed network was generated using the (protein)–protein–chemical interaction web tool STITCH 3.1 beta (Kuhn et al. 2009). Associations with a confidence score of ≥0.4 (medium level) were displayed. In supplementary Table S3, the type and confidence level of each association are listed

According to GeneDecks analysis, chemical compounds with immune-modulating properties, eicosanoid, Vitamin D3 (1,25-dihydroxyvitamin D3) and retinoic acid (RA) showed an association with genes regulated by *L. plantarum* 299v (Table S2). The enzymes prostaglandin reductase 1 (PTGR1) and adenosine deaminase (ADA) were down-regulated in this study. PTGR1 catalyses the conversion of the eicosanoid leukotriene B4 to its biologically less active metabolite 12-oxo-leukotriene B4. ADA catalyses breakdown of adenosine to inosine and NH3 in the purine pathway (called significant in this study; see Table S2) and plays a role in neurotransmission, the development of the immune system and differentiation of epithelial cells and monocytes (Moriwaki et al. 1999). Deficiency in ADA causes severe combined immunodeficiency disease (SCID). Together with the genes/enzymes in question, all these compounds were uploaded in STITCH to establish a protein–chemical interaction network (Fig. 4). In supplementary Table S3, the type and confidence level of associations between components of this network are provided. Both 1,25-dihydroxyvitamin D3 and PPARG play a pivotal role in this network. 1,25-dihydroxyvitamin D3 can down-regulate the expression of PPARG in adipocytes (Kong and Li 2006) and stimulate the expression of CA2 (Biskobing et al. 1997) and CXCR4 (Biswas et al. 1998). Furthermore, the enzyme that initiates degradation of biological active 1,25-dihydroxyvitamin D3, CYP24A1, was down-regulated in response to *L. plantarum* 299v.

## Discussion

In most clinical trials with human cohorts, beneficial effect of *Lactobacilli* intake is recorded after several weeks of intake of a fixed dose before each meal. To ensure that all piglets received a similar volume of the *L. plantarum* suspension, we delivered it directly in the oral cavity using a syringe. However, administration in the oral cavity of young pigs in a comparable frequency as used in human trials (e.g. 3 times a day for several weeks) would be too stressful for young pigs. Therefore, we started with a single high dose of *L. plantarum* 299v followed by three consecutive daily dosings 10 days later. Despite this different scheme of intake, information gained in this study in pigs might be valuable for understanding of the mechanism of action of this probiotic bacterium in parts of the human intestine (jejunum and ileum) that can only be sampled in using surgery.

Modulation of gene expression was observed mainly in the ileum, the immunologically most active part of the intestine of pigs and of clusters of genes involved in specific immunological (or immune-related) pathways/processes characteristic for the ileum and also described in previous studies in which the transcriptional response in the intestine was measured after consumption of *L. plantarum* and other probiotic bacteria. QRT-PCR analysis indicated that for particular genes, a considerable natural variation in expression levels of genes in the ileum exists between individual pigs. This difference in biological/genetic background might have influenced the response to *L. plantarum* 299v. However, identification of meaningful pathways retrieved in this study suggests that 8 pigs per group/pool were sufficient to allow for the detection of significant effects despite this natural variation.

Inflammatory mediators like IL8, IL1B, FOS, NFKB and acute-phase response proteins like REG3A, for which up-regulation of expression was observed in several animal studies in which the pigs intestine was challenged with bacterial pathogens like ETEC and Salmonella (Niewold et al. 2010; Hulst et al. 2013, and reference herein), did not responded at all to *L. plantarum* 299v (see also Fig. 2; IL1B expression). This clearly showed that the intestinal mucosa of pigs responded differently, and less vigorous, to exposure to *L. plantarum* 299v bacteria than to hostile enteric bacteria. The fact that *L. plantarum* 299v did not colonize in the pig’s intestine may be related to this mild response.

In agreement with other in vivo studies conducted with *L. plantarum* strain WCFS1 in humans (Bujalance et al. 2007; Troost et al. 2008; van Baarlen et al. 2009), our results also indicate that *L. plantarum* regulates the influx and/or enhances the proliferation and differentiation of lymphocytes in the intestine without inducing NFKB-mediated inflammatory processes. According to our predictions, and indicated by up-regulation of cell-specific marker genes, gene expression in CD4+ lymphocytes, CD14+ monocytes, adipocytes and B cells were regulated in response to *L. plantarum* 299v. The latter cells can be progenitor B cells that proliferate to IgA-plasma cell progenitors in the Peyer’s patches. With respect to this B cell proliferation, similar observations have been described in studies in which mice and humans were administrated with other probiotic bacteria (Fukushima et al. 1998, 1999; Tsai et al. 2010). However, *L. plantarum* 299v could also stimulate an influx of CD14+ monocytes and B cells from the periphery to the ileum.

Cell wall component teichoic acid (TA) was identified as a dominant MAMP of gram-positive bacteria including several probiotic *Lactobacillus* bacteria (Lebeer et al. 2010). Recognition of *L. rhamnosusGG* TA’s by TLR2/6 dimers results in NFKB-mediated cytokine production (Claes et al. 2012). In this study, only up-regulation of TLR9 expression was observed, which recognizes bacterial CpG DNA, but no regulation of TLR2/6 gene expression. A recent study, in which healthy non-compromised mice were administered with a mutant of *L. plantarum* strain WCFS1 containing much less d-alanine incorporated in its cell wand TA, indicated that this dominant MAMP not only influenced a pro-inflammatory response, but also a systemic anti-inflammatory response characterized by an increase in regulatory T cells (Treg) in the spleen (Smelt et al. 2013). Interestingly, the gene adenosine deaminase (ADA) was found down-regulated more than tenfold in our study. Extracellular adenosine produced by Tregs acts as anti-inflammatory mediator on the immune system. A recent study indicated that accumulation of adenosine produced by Tregs in ADA-deficient SCID patients contributes to autoimmune manifestations (Sauer et al. 2012). Therefore, *L. plantarum*-induced down-regulation of ADA in the ileum of pigs and increase in splenic Tregs in mice (Smelt et al. 2013) suggest that adenosine-mediated immune suppression by Tregs may be initiated in the intestines and transmitted to the periphery. Further studies in non-compromised animals with probiotic bacteria with altered TA structures on their surface focusing on this ADA-Treg immune mechanism could reveal valuable information about how probiotic bacteria communicate their health beneficial effects to other parts of the host body.

By building a network of transcription factors, we identified a set of B cell-specific transcription factors (BCL6, BCOR, SPIB, SPI1, TCF4 and IKZ3). Up-regulation of BCOR may suppress BCL6-mediated transcription in order to prevent excessive B cell maturation. In addition, down-regulation of CCL28 and IgA-transporter PIGR may also counteract excessive B cell migration and unnecessary secretion of produced IgA into the lumen, respectively.

NFKB-mediated transcription can be repressed by up-regulation of CLU, a complement cytolysis inhibitor (Takase et al. 2008) and several ubiquitin-specific proteases, like USP48 (Tzimas et al. 2006) and USP21 (Xu et al. 2010). In addition, genes like DMBT1 (a target gene for the intracellular pathogen recognition receptor NOD2), AGT, CFB and BPI (bactericidal/permeability-increasing protein), normally found up-regulated in intestine infected by bacterial pathogens, were all down-regulated after *L. plantarum* administration. Furthermore, ubiquitin-conjugating enzymes UBE2E2, UBE2V2 and UBE2T were up-regulated. In conjunction with anti-apoptotic c-IAPs (like BIRC5; up-regulated in this study), human UBE2s promote polyubiquitination of RIPK1 and suppresses DDX58 (alias RIG-I) ubiquitination by RNF125 (Dynek et al. 2010). Both cytosolic receptors, RIPK1 and DDX58, are essential for recognition of viral and bacterial antigens and subsequent activation of interferon regulatory factors (IRF’s) or NFKB-mediated transcription of chemotactic and pro-inflammatory factors like IL1B.

Down-regulation of the enzyme HSD11B2, which catalyses the inter-conversion of the glucocorticoids (GCs) cortisol and cortisone, and the regulation of several other corticosteroid-related genes (SGK2, OSBPL3, OSBPL8, SOAT1 and AKR1C1) suggest that *L.plantarum* 299v also affected the concentration of endocrine GCs in the ileum. GCs are potent inhibitors of inflammatory cytokine production by macrophages. Interestingly, in the promoter of porcine MIF (macrophage migration inhibitory factor), two potential PPARG binding sites were identified recently (Chen et al. 2010). MIF antagonizes the inhibition of GC-induced cytokine production in macrophages by restoring the transcriptional activity in particular of NFKB. Therefore, down-regulation of PPARG and/or modulation of its transcriptional activity by NRIP1, in conjunction with regulation of GC levels, could be one of the mechanisms of *L. plantarum* 299v to control the inflammatory activity of macrophages in the ileum.

NRIP1-regulated PPARG signalling has also been shown to affect lipid metabolism and differentiation of adipocytes (Debevec et al. 2007). Our bioinformatics analysis predicted that most of the response genes related to PPARG signalling and lipid/steroid metabolism are expressed by adipocytes and CD14+ monocytes. White adipose tissue is found in the sub-mucosa, a layer just underneath the lamina propia that surrounds the Peyer’s patches embedded in the mucosal layer. Crosstalk between cultured macrophages and adipocytes after exposure to heat-killed *L. gasseri* and *L. rhamnosus GG* resulted in down-regulation of PPARG, and subsequently, to an altered cytokine response in macrophages (Miyazawa et al. 2012). A similar mechanism of crosstalk between macrophages and adipocytes in the sub-mucosal layer of the ileum of pigs can also direct polarization of macrophages towards an anti-inflammatory phenotype (Odegaard et al. 2007; Lumeng et al. 2007).

According to Fig. 4, 1,25-dihydroxyvitamin D3 metabolism may play a key role in crosstalk between the different types of cells in the ileum that respond to *L. plantarum* 299v. Endogenous 1,25-dihydroxyvitamin D3 can block expression of the transcription factor/regulators C/EBP-alpha, sterol regulatory element-binding protein-1 and PPARG in adipocytes, suppressing cell differentiation in these cells (Wood 2008). Production of 1,25-dihydroxyvitamin D3 by human B cells is stimulated by activation of the CD40 receptor and regulated by a complex feedback mechanism in which 1,25-dihydroxyvitamin D3 itself, transient-receptor-potential-cation-channels (like TRPV6; down-regulated here) and the enzyme that degrades 1,25-dihydroxyvitamin D3 (CYP24A; down-regulated here) are involved (Heine et al. 2008).

The inflammatory mediator leukotriene B4 is produced by polymorphonuclear leucocytes and macrophages. A previous studies indicated that leukotriene B4 can activate T cells that inhibit Epstein-Barr virus-induced proliferation of cord blood-derived B-lymphocytes (Liu et al. 2008) and that living and heat-killed preparations of lactic acid bacterial strains were able to inhibit leukotriene B4 production in murine macrophages (Kimoto-Nira et al. 2009). In the current study, we observed a moderate down-regulation (twofold) of PTGR1 (alias LTB4DH), the enzyme that catalyses the conversion of inflammatory mediator leukotriene B4 to biologically less active 12-oxo-leukotriene B4, suggesting that the activity of leukotriene B4 was raised in response to *L. plantarum* 299v. This is in contrast with results of two in vitro studies cited above and may suggest that in vivo regulation of leukotriene B4 metabolism in the ileum may be dependent on additional factors and/or specific types of cells not present in the above used in vitro cell systems.

Calves treated with corticosteroids produced low levels of IgA in the intestine (Husband et al. 1973). Also, human asthma patients treated with inhaled corticosteroids produce less salivary IgA (Fukushima et al. 2005). Furthermore, studies in ferrets indicated that development of Peyer’s patches and/or delay in the migration of IgA-plasma cell progenitors from this lymphoid organ into the lamina propia was depressed by corticosteroids (Carlile and Beck 1983). This suggests that besides 1,25-dihydroxyvitamin D3 and leukotriene B4, steroids may also play a role in the crosstalk between cells that respond to *L.plantarum* 299v.

In conclusion, our data suggested that the probiotic bacterium *L. plantarum* 299v regulates the activity of adipocytes and/or different subsets of B cell in the ileum of pigs. Either infiltration of the ileum by these cells or their maturation/differentiation to functional cells is stimulated. Progenitor B cells may mature to IgA-committed plasma cells and travel from the Peyer’s patches to the lamina propia in response to *L. plantarum* 299v. In case external challenges are detected, these IgA-producing cells can respond directly by secretion of IgA antibodies into the lumen at the site of damage. In addition, in this study, induced repression of NFKB-mediated transcription and PPARG signalling may implicate that *L. plantarum* 299v has a prophylactic effect on development of inflammation in the ileum of pigs. Crosstalk between specific immune cells present in the ileum and sub-mucosal adipocytes may play an important role in this repression. The role of leukotriene B4, 1,25-dihydroxyvitamin D3, steroids and adenosine in this crosstalk is an interesting issue for further research.

## Electronic supplementary material

Supplementary material 1 (XLSX 83 kb)
